# Intimate partner physical violence among women in Shimelba refugee camp, northern Ethiopia

**DOI:** 10.1186/1471-2458-12-125

**Published:** 2012-02-13

**Authors:** Girmatsion Feseha, Abebe G/mariam, Mulusew Gerbaba

**Affiliations:** 1 Department of Nursing, College of Health Sciences, Mekelle University, Mekelle, Ethiopia; 2 Department of Population and Family Health, College of Public and Medical Sciences, Jimma University, Jimma, Ethiopia

**Keywords:** Intimate partner violence, Risk factors, Refugee camp, Ethiopia

## Abstract

**Background:**

Domestic violence has unwanted effects on the physical and psychological well-being of women, which have been recognized globally as an important public health problem. Violence perpetrated by intimate partner is one form of domestic violence, a serious human rights abuse and a public health issue, among refugees owing to its substantial consequences for women's physical, mental and reproductive health problems. Because the incidents are under-reported, the true scale of the problem is unknown and unexamined among refugee women in Ethiopia. Thus, this study aim to assess the magnitude of intimate partner physical violence and associated factors among women in Shimelba refugee camp, Northern Ethiopia.

**Methods:**

A community-based cross-sectional study was conducted among a sample of 422 refugee women from March to April 2011. A simple random sampling method was used to select the study subjects from seven zones of the refugee camp. Census was done to identify all households with women having an intimate partner. A pre-tested interviewer guided structured questionnaire was used for data collection. Data were entered, cleaned and analyzed using SPSS software version 16.0. Descriptive, bivariate and multivariate logistic regression analyses were done where applicable. A p-value less than 0.05 with 95% CI were set and used as a cut-off point to examine the statistical association between the explanatory and outcome variables.

**Results:**

The prevalence of physical violence in the last 12 months and lifetime were 107(25.5%) and 131(31.0%) respectively. The commonest forms of physical violence reported included slapping 101(61.6%) and throwing objects 32(19.5%). Significant risk factors associated with experiencing physical violence were being a farmer (AOR = 3.0[95%CI: 1.7, 5.5]), knowing women in neighborhood whose husband to beat them (AOR = 1.87[95%CI: 1.0, 3.5]), being a Muslim (AOR = 2.4 [95%C.I: 1.107, 5.5]), and having a drunkard partner (AOR = 2.1[95%C.I:1.0, 4.5]).

**Conclusions:**

Intimate partner physical violence was found to be high and a serious problem among women in Shimelba refugee camp. Multifaceted interventions such as male counseling, increasing awareness on the consequences of intimate partner violence and the effect of substance use like alcohol will help to reduce intimate partner violence.

## Background

Violence against women is an important public health concern owing to its substantial consequences for women's physical, mental and reproductive health problems [1]. Consequences of domestic violence, characterized by women's experience of physical, psychological, and sexual injury or threat are manifold. A significant number of studies consider domestic violence as risk factor for health problems, including injury and death [2,3]. Intimate partner violence is the most common form of violence against women and an important cause of morbidity and mortality [4]. The most common forms of violence against women are physical, sexual, and emotional abuse by husband's or intimate partner. A survey indicated that 10 to 58% of women have experienced physical abuse by an intimate partner in their lifetimes [5].

Violence against women is usually targeted at women and girls due to their unequal treatment nature in society. It can takes place in the home, on the streets, in schools, in the workplace, in farm areas, refugee camps which is perpetrated by persons in positions of power [6,7]. Wife beating, the most widespread form of domestic violence, has adverse consequences on the health and wellbeing of women and is a major cause of disability and death in many countries. In the past few years, it has been widely reported in developing countries' contexts, where patriarchal family norms are common and patriarchal gender relations were reinforced by traditional cultural, legal, and perhaps religious legacies [8,9].

There are many factors exacerbating the persistence of violence against women. The exclusion of women and girls from the public arena increases their vulnerability to violence within the family, reinforcing gender-based discrimination and keeps women subordinate to men or their partner [10]. Violence against women is not only a manifestation of sex inequality, but also serves to maintain this unequal balance of power. In some cases, perpetrators consciously use violence as a mechanism for subordination. For example, violence by intimate partners is often used to demonstrate and enforce a man's position as head of the household or relationship. According to World Health Organization (WHO), violence is the result of the complex interplay of individual, relationship, social, cultural and environmental factors. The environment and social norms are the factors that may condone violence; however, are examined and reported infrequently [11].

Recent experiences showed that violence against women is the most common problem among women in refugee camps. It is a complex problem that cannot be attributed to a single cause but to a diverse set of factors, including demographic, socioeconomic and cultural ones [6].

Refugee women are being victimized twice, in their lives, was disrupted due to the conflict and then their husbands in the camps subjecting them to another form of violence [12]. Yet it has received little attention, as sexual and gender based violence (SGBV) policy and practice focuses on other forms of violence. Because incidents of sexual and gender-based violence are under-reported making the true scale of the problem is unknown [12,13].

Despite its increasing global importance, there has been little research on domestic violence against women in Ethiopia. In Ethiopia, even the existing literatures only depicts the prevalence and characteristics of intimate partner violence against women among population found in our communities but, data related to refugee population is limited however; there are evidences that indicate the pervasiveness of the problem. Thus, understanding the magnitude of the problems and the reasons behind intimate partner violence among refugee women is crucial for program planer to design effective preventive strategies.

## Methods

### Study setting

This cross sectional study was conducted from March to April 2011 in Shimelba refugee camp, found in Tigray regional state, seventy kilometers from the Eritrean border and 1300 kilometers from Addis Ababa. Shimelba refugee camp was established in 2000 and hosts people who fled from Eritrea during the Ethio-Eritrean border conflict of 1998-2000. During the mid-December of 2009, there were 13, 943 Eritrean refugees mainly from the Tigrigna, Kunama and Saho ethnic groups in the study area. The Administration for Refugees/Returnees Affairs reported that about 1,917 women aged between 15-49 years old were living with their partners [13].

There are seven zones inside the camp administratively led by the Ethiopian government in collaboration with United Nation Higher Commission for Refugee. Each of the seven zones consists of 274, 308, 264, 251, 211, 343, and 274 women aged 15-49 years old respectively. There is a "central committee" elected by the residing population inside the camp, and the committee represents the refugees on various issues liaising with Non Governmental Organizations and Ethiopian government. Newcomers are expected to construct their own shelter by using locally available material. Refugees are not allowed to work outside the camp. As a result, fewer than 10% are employed.

There is no other means of generating income for the women though there are small numbers of beauty salons, restaurants, and retail shops and some refugees have been hired to work at these places. Other refugees have found employment with United Nation Higher Commission for Refugee's implementing partners. When refugees arrive in the camps, they are registered and issued with ration cards. These cards are used as their identification and also for the purpose of food distribution which is provided by United Nation Higher Commission for Refugees in collaboration with World Food Program [14].

### Study design and sampling

A community based cross sectional study using quantitative method of data collection was employed. All married or cohabiting women found in Shimelba refugee camp during data collection period were recruited as a source population for the study. The study population were a randomly sampled married/cohabitated women residing in camp and those who are living with their partner for at least one year preceding the survey. Census was conducted to select all households in the camp with married women or women who were partnered or cohabited. Households were selected as study unit using simple random sampling method after enumeration and coding was carried out. In case where two or three pairs of partners' were living in the same house a sub number was assigned by taking one reference women with partner and another sub number was given for women with partners' and their name was included with sub number then sampling frame was created.

The sample size was calculated using a single population proportion formula. Since there was no previous study describing on the prevalence of physical violence among married women in study area, to get a maximum sample size we assumed 50% prevalence of intimate partner's physical violence with 95% confidence interval and 5% degree of precision. 10% was added for non response and the final calculated sample was 422. The sample size was proportional allocated to the size of the seven zones. Three repeated visits were attempted in the absence of selected subject at their house.

### Measurements

Data were collected using close ended structured questionnaire adapted to local context by reviewing similar literatures done in other refugee camps elsewhere to address the objectives of the study. To keep further validity and make the findings comparable measurement of physical violence was adopted from WHO questionnaire on domestic violence and intimate Partner Violence [15-19]. Five percent of questionnaires were pre tested to check for consistency, coherence and amended accordingly. The questionnaire contains variables on socio demographic information, the experience of physical violence; family and husband related history. Ten female high school graduates who can speak the local languages (Tigrigna and Kunama) collected the information by going from house to house. Three supervisors were also recruited based on their previous field experience with household surveys. Three days intensive training was given to the data enumerators on how to collect data, when and how to make an interview and about ethical issues, emphasizing on the importance of safety of participants and data quality. To get the outcome of interest lifetime and 12 months experience of intimate partner physical violence was enquired. Specific acts such as throwing objects; pushing, grabbing or shoveling; slapping; punching with fist or something else that can hurt; kicked, bit or hit; hit or tried to hit with something; beat up; chocking; burning or scalding; threatening and/or attacking with a knife, gunning, or other type of weapon were operationally defined as an act of physical violence. Women who reported that their husbands/partners have ever perpetrated at least one of these acts were reported to have experienced physical violence.

Regular supervision of data collection was made by investigators and supervisors. The questionnaires were checked for completeness and consistency and then data were edited, coded, entered, cleaned and analyzed using SPSS software version 16.0. Descriptive analyses such as frequencies, percentages, tables, figures were used to display the results. Bivariate logistic regression was done between each independent variable and outcome of the interest to assess statistical association. Multiple logistic regressions were performed to identify the most significant predictor of physical partner violence and to control for confounders.

Odds ratio and confidence interval with 95% confidence limits and significance level (*P *< 0.05) was used to determine level of significance. The prevalence of physical violence was estimated for two time frames: the 12 months preceding the interview and any time during the woman's life from the time she started relationship with the current intimate partner.

Ethical clearance was obtained first from College of Public Health and Medical Sciences Ethical Review Board, Jimma. In addition, official permission was taken from Administration for Refugees/Returnees Affairs (ARRA) from Addis Ababa and Zonal Office in Shire and finally cooperation letter was written to Shimelba refugee camp to undertake the study. The importance of the study was explained to each respondent, verbal consent was obtained and assurance was given about the confidentiality of the responses taking in to account the guidelines on ethical and safety recommendations for research on domestic violence against women [20].

## Results

### Socio-demographic characteristics

All the sampled 422 women responded to the questionnaire making a response rate of 100%. Out of this, 273 (64.7%) came from rural origin, Eighty six (20.4%) had previous history of living in other refugee camp. Their mean age was 26.2(SD ± 6.33) years. One hundred three (41%) were illiterate and 256 (60.7%) are married. Regarding their occupational status 371(87.9%) were housewives while 9 (2.1%) are employed. Fifty five (13.0%) of them reported that their partner had other wife previously. Most of women 381(90.3%) have stayed in the camp for more than one year. The mean duration of marital lifetime was 6.15 years (SD ± 5.2) (Table 1)

**Table 1:** Socio-demographic characteristics of women with partners' (n = 422), Shimelba refugee camp, April 2011

Socio-demographic Variables	Number	%
Age		
15-19	40	9.5
20-24	156	37
25-29	123	29.1
30-34	54	12.8
> 35	49	11.6
Religion		
Orthodox	266	63
Muslim	68	16.1
Catholic	54	12.8
Protestant	29	6.9
Other*	5	1.2
Ethnicity		
Tigrigna	187	44.3
kunama Eritrea	217	51.4
kunama Tigray	2	0.5
Saho	10	2.4
Others**	6	1.4
Occupation		
Housewife	371	87.9
Trading	12	2.8
Employee	9	2.1
Farmer	24	5.7
Other***	6	1.4
Educational status		
Illiterate	173	41
Can read and write	44	10.4
Primary school(1-8 grade)	133	31.5
High school(9-12 grade)	64	15.2
Higher education	8	1.9
Type of current partner		
Husband	256	60.7
Co-habited (live together in the same house without formal marriage)	118	28
Boyfriend	48	11.4
Duration of relationship		
< 2 years	49	11.6
2-5 years	202	47.9
> 6 years	171	40.5

* Others include: - Jehovah, Pagan

** Others include: - Tigre, Amhara (Ethiopian)

***Others include: - barberry, women who have personal ability and preparing decoration for house, "Tela" seller

### Prevalence of intimate partner physical violence

**Figure 1:**
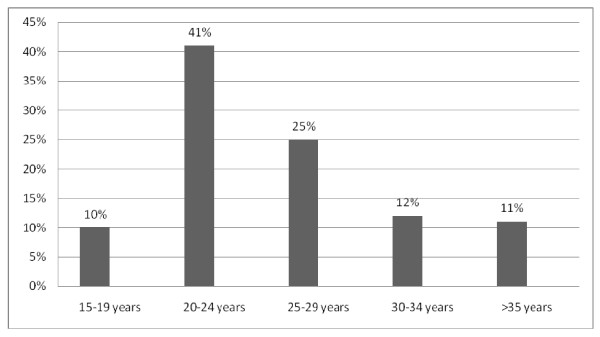
**Magnitude of partner's physical violence with the age categories of the women in the last 12 months among the women in Shimelba refugee camp, 2011**.

Almost two thirds of the respondents 168 (38.9%) had interpersonal conflict such as disagreement, physical or psychological with their partner since their marriage relationship or relationship time. Out of this 131(78.9%) had a history of physical violence with their partner. Of these 90 (54.9%) of them had experienced this once in four months, 64(39.0%) once in three months and 10(6.1%) daily of the physical violence experience within their marriage. The life time prevalence of any form of physical violence was 131(31.0%). One out of every four women 107(25.35%) were physically abused in the last 12 months. Sixty (56.0%) of women whose partner had history of drinking alcohol were physical abused in the last 12 months. Regarding their experience of different types of physical violence, slapping was the most frequently reported act of violence accounting for 101(61.6%) of the lifetime experience and 78(47.6%) in the last 12 months (Table 2). Intimate Partner violence was found to contribute to health and health related consequences of the women face to difficulty with daily activity 63%, pain 23.5%, difficulty during walk 14.8% and fracture or dislocations, 2.5% damage to ear as result of slapping, 2.5% deep cut of body parts and psychological disturbances. Out of the studied subjects currently pregnant women were found to be victims of physical violence. Among those pregnant women the lifetime and 12 months prevalence of intimate partner physical violence were 17(30.4%) and 11(19.6%) respectively (Table 3). In this study most of the abused women in the last 12 months were found in the age group 15-24 years (Figure 1).

**Table 2:** Prevalence of physical partner's violence in the life time and 12 months among women in Shimelba refugee camp, 2011

Type of physical violence	Life time prevalence	12 month prevalence	
	
	Yes (%)		Yes (%)
Threw objects	32(19.5)		16(9.8)
Push, gripped/shoved	11(6.7)		7(4.3)
Slapped	101(61.6)		78(47.6)
Kicked/hit/bit	19(11.6)		12(7.3)
Hit with something /try to hit	10(6.1)		4(2.4)
Beat up	4(2.4)		1(0.6)
Choked	5(3.0)		1(0.6)
Threatening with knife or weapon	3(1.8)		1(0.6)
Used knife/weapon	2(1.2)		0
Overall prevalence of physical partners violence	131(31.0)	-	107(25.3)

**Table 3:** Life time and 12 months prevalence of physical partner's violence among currently pregnant women in Shimelba refugee camp, 2011

Type of physical partner violence among currently pregnant women	Life time prevalence	12 month prevalence
	
	Yes (%)	Yes (%)
Threw some thing	9(28.1)	2(12.5)
Push, gripped/shoved	2(18)	2(28.6)
Slapped	9(8.9)	5(6.4)
Kicked/hit/bit	4(21.1)	2(16.7)
Hit with something /try to hit	1(10)	-
Choked	2(40)	-
Overall prevalence of physical partners	17(4.0)	11(2.6)

### Factor related to life time intimate partner physical violence

After controlling for all significant variables in the bivariate analysis, multivariable logistic regression showed participants who were Muslim by religion were two times more likely to experience lifetime physical violence as compared to those who were Christians (OR = 2.5 [95% C.I: 1.1, 5.5]), women who were farmer were 13 times more likely to have experienced IPV compared to the housewives (OR = 13.0 [95%C.I: 3.7, 45.5]), women whose mothers had experienced violence were nine times more likely to experience intimate physical violence compared to those women who had no history of maternal IPV (OR = 9.7[C.I: 5.2, 18.1]) and the risk of physical violence among women who knew of other women in neighborhood who had experienced IPV was higher compared to those who did not. The odds of experiencing physical violence in their life time was five times more among women whose current husband was illiterate and can read and write compared to those women whose current partners have higher level of education (OR = 5.3[95%C.I: 1.1, 24.0]) and (OR = (5.4 [95%C.I: 1.1, 26.3]) respectively.

Physical violence is 52.0% less among women who have daily laborer partner by occupation than those who have farmer partner (OR = 0.4[95%C.I: 0.2, 0.9]). Women attended primary and secondary schools were less likely to experience physical violence in their life time (by 78.0% times) and (by 85% times) than those who have diploma and above (OR = 0.2[95%C.I: 0.5, 0.9]) and (OR = 0.1[95%C.I: 0.03, 0.72]) respectively.

After controlling for all significant variables in the bivariate analysis, multivariable logistic regression showed participants who were Muslim by religion were about two times more likely to experience physical violence in lifetime as compared to those who were Christian followers (OR = 2.5 [95%C.I: 1.1, 5.5]), about 13 times more higher for those farmer women as compared to those who were housewife by occupation (OR = 13.0 [95%C.I: 3.7, 45.5]), women who had history of maternal intimate partner violence were nine times more likely to experience intimate physical violence compared to those women who had no history of maternal intimate partner violence (OR = 9.7[95% C.I: 5.2, 18.1]) and the risk of physical violence among women who know other husband to beat his wife in neighbor was high compared to those who don't know (OR = 1.8[95%C.I: 1.0, 3.5]) (Table 4).

**Table 4:** Factors related to experiencing of partners' physical violence in life time among women in Shimelba refugee camp, 2011

Variable	Physical violence in the life time		Odds Ratio and 95% C.I	
			
	No (%)	Yes (%)	Adjusted (AOR)**	P value
**Religion of women**				
Orthodox	200(75)	66 (50)	**1**	
Muslim	33(11)	35 (27)	2.4(1.1, 5.5)*	**0.027**
Catholic	32(11)	22 (17)	1.2(0.5,3.04)	
Protestant	22(8)	7(5)	1.2(0.42,3.90)	
Others*	4(1)	1(1)	0.4(0.03, 6.0)	
**Occupation of women**				
Housewife	266(72)	107(80)	1	
Trading	8(3)	4(3)	0.5(0.1, 2.1)	
Employee	6(2)	3(2)	3.2(0.4, 23.5)	
Farmer	5(2)	19(15)	13.0(3.7,45.5)*	**< 0.001**
Women know other husband to beat his wife*				
Yes	112(38)	92(70)	1.8(1.0, 3.5)*	**0.049**
No	179(62)	39(30)	1	

Others*: - Jah^.^weh, Pagan, *presence of conflict in their neighbors

### Factors related to intimate partner violence in the last 12 months

In bivariate analysis the likelihood of experiencing intimate partner physical violence in the last 12 months was higher among Muslim and Catholic religion follower than those Orthodox Christian followers (OR = 2.8[95%C.I: 1.6,5.0]) and (OR = 2.4 [95%C.I: 1.2, 4.5]) respectively.

There is also higher likelihood among farmer women by occupation than those housewives (OR = 5.6[95%C.I: 2.3, 13.2]). And also the likelihood of intimate partner physical violence was higher among women whose current relationship with partner was arranged by couple's agreement than those families supported relationships (OR = 2.0[95%C.I: 1.3, 3.3]).

Those women whose partner was employee (either governmental or nongovernmental) were less likely to have physical violence than those women whose partner was farmer (OR = 0.3[95%C.I: 0.1, 0.8]). Again the likelihood of women whose current partners 'drink alcohol and chew 'khat' or smoke cigarette or tobacco to experience intimate physical violence were higher than those women's partner don't drink alcohol and chew khat or smoke cigarette or tobacco(OR = 1.9[95%C.I: 1.2, 3.0]) and (OR = 3.6[95%C.I: 2.0, 6.2]) respectively. However, there was less likelihood of experiencing intimate partner physical violence in the last 12 months among women that could read and write, attend primary and secondary school as compared to women in higher education level. In addition, those women whose partner was above grade 12 have less likelihood of physical violence than those women have illiterate partner (OR = 0.1[95%C.I: 0.01, 0.9]).

After adjusting for all significant variables, the multiple regression analysis showed women who had history of maternal intimate partner violence were six times more likely to experience intimate partner physical violence in the last 12 months compared to women who had no history of maternal intimate partner violence (OR = 6.7[95%C.I: 3.2, 14.3]). The odds of women whose current partners drink alcohol were 2 times more likely to have physical violence than those women having partner not drink alcohol (OR = 2.1[95% C.I 1.04, 4.5]) but, those women whose partner was employee were 93% time less experience of physical violence compare to those women whose current partner is farmer (OR = 0.07[95%C.I 0.1, 0.41]) (Table 5)

**Table 5:** Factors related to experiencing of partners' physical violence in the last 12 months among women in Shimelba refugee camp, 2011

Variable	Physical violence in last 12 months		Odds Ratio and 95% C.I	
	
	No (%)	Yes (%)	Adjusted (AOR)**	P value
**Occupation of partner**				
Farmer	86(27)	41 (38)	1	
Merchant	30(10)	16 (15)	0.9(0.2,3.4)	
Employee	33(10)	5 (5)	**0.07(0.1, 0.4)**	**0.003**
Daily laborer	63(20)	14 (13)	0.39(0.13,1.21)	
No job	92(29)	29 (27)	**0.21(0.06, 0.7)**	**0.017**
Other *	11(3)	2 (2)	1.6(0.2, 12.5)	
**Partner drink alcohol**				
Yes	124(39)	60 (56)	**2.1(1.04, 4..5)**	**0.038**
No	191(61)	47 (44)	1	
**Injury as result of violence**				
Yes	16(5)	65(61)	**37.4(14.4, 97.4)**	**< 0.001**
No	299(95)	42(39)	1	

*Others include: - barberry, women who have personal ability and preparing decoration for house, "Tela" seller

## Discussion

In this study almost two fifths of the respondents had interpersonal conflict with their partner since their marriage or relationship time, out of which one third had a history of physical violence. The lifetime prevalence rate of intimate partner physical violence was 31.0% which was similarly reported in the study done in India 30% [19], and Gondar (32.2%) [21], and lower than the study reported from Palestinian refugees (42.5%) and the Jordanian refugee camps (44.7%) [22], even lower than the study done among married women in rural Ethiopia which was 49.5% [16].

This differences could be due to the fact that their marital relationship is arranged by themselves without family involvement shoed there is statistical significant difference between self arranged relation and experiencing of physical violence than family supported which was observed to be statistically significant. The differences could also ascribe to underreporting and cultural make up of family formation patterns. This variation can also be attributed not only to the differences in the levels of violence between settings, but also to differences in research methods, definitions of violence, sampling techniques, interviewer training and skills, and cultural differences that affect respondent's willingness to reveal intimate experiences. For these reasons, it is difficult to make direct comparisons or to make judgments between cultures or countries about in which society intimate partner violence is worst [23].

The prevalence of intimate partner physical violence in the last 12 months found in the present study was consistent with the study done in Leon, Nicaragua (27%) and lower when compared to study conducted in the Republic of Korea (38%), and Palestinian women in the West Bank and Gaza Strip (52%) [24]. Apart from methodological issues, the difference in magnitude of physical violence reported in different literatures and this study could be explained by socio cultural and societal perspectives and contexts of the population under study that differ between the nations.

The chance of experiencing physical violence in life time in this study was significantly associated with injury and multiple logistic regression showed that those women who are victims of partner violence were 37 times more likely to suffer injury in the last 12 months (AOR = 37.4, [95% C.I 14.4, 97.4]), which is consistence with other one study conducted elsewhere [25], and also consistence with studies in Canada and the United States, which showed that female victims of partner violence are three times more likely to suffer injury, five times more likely to receive medical attention [26]. Physical violence in the last 12 months was 2 times higher among women that the current relationship with their partner was arranged by couples agreement than those family supported relation (OR = 2.0 [95%C.I:1.2, 3.2]). After adjusted the risk is increased among women who know that their current partner has other wife than those that their current partner did not have other wife (AOR = 1.9[95%C.I: 1.0, 3.6]). This is consistence with study done by James K.et al. in Uganda the husband having another partner (OR = 2.4, 95% CI 1.0, 5.7) were associated with higher risk of intimate partner violence.

In multiple logistic regressions analysis level there is higher likelihood of experiencing physical violence in the lifetime among Muslim than those Orthodox Christian followers women (AOR = 2.4 [95%C.I: 1.1, 5.5]) Which is consistence with study done in Arsi showed significant difference between Muslim and Orthodox Christian [27], the difference could be due the presence of most Saho ethnic and Kunama ethnics are Muslim in addition to this they were from rural origin and came from low social status. After controlling for other variables being experiencing physical violence in the last 12 months was 2 times higher among women whose current partners 'drink alcohol than those women's partner don't drink alcohol (AOR = 2.1[95%C.I: 1.0, 4.5]) this is consistence with study conducted around Gondar that physical violence was about five times more likely to occur among women whose male partners consume alcohol frequently and study in Aris zone, use of alcohol were associated with intimate partner physical violence [21,27]. A Cross sectional study in Albania indicated risk of violence reduced with decreasing employment status of women: women in blue collar work, housekeepers, and unemployed women were at lower risk than those in white collar occupations [28] which is different from the finding from this study that farmer women were more likely to experience intimate physical violence as compared to those housewife (OR = 5.6 [95%C.I: 2.3, 13.2]). This might be due to the difference in study population, sampling proportion, techniques to collect the sample and the socio cultural context where dominated by patriarchal belief for male dominance.

In this study it was less likely to experience physical violence in the last 12 months among women that can read and write, completed, primary and secondary school as compared to women in higher education level. It is less by 88.5% times among women whose partner was above grade 12 than women having illiterate partner (OR = 0.1[95%C.I: 0.0, 0.9]) and less by 68% times among the women whose partner was employee than those women whose partner was farmer (OR = 0.3[95%C.I: 0.1, 0.8]). This is similar with study done in Nicaraguan the risk increased with increasing educational level for women, with decreasing educational level for men [29] and study in Bangladesh in 2005 husband's education beyond the 10th grade decreased the risk of violence [30].

Those farmer women were 13 times more likely to experience physical violence in lifetime as compared to housewife (AOR = 13.0 [95%C.I: 3.7, 45.5]) and those women whose partner was employee were 93% time less experience of physical violence in the last 12 months as compare to those women whose current partner is farmer (AOR = 0.07[95%C.I 0.1, 0.4]). This finding was consistent with study done in South Africa factors include social and demographic characteristics of the men and women, their economic circumstances influence violence against women [31].

Similarly study in south Asia in 2003 showed financial dependence on a partner or unemployment were the risk factors that contribute to this phenomenon [32]. Similarly study in South Asia in 2003 shown financial dependence on a partner or unemployment were the risk factors that contribute to this phenomenon [33]. After controlling for other variables being experiencing physical violence in the last 12 months was 2 times more higher among women whose current partners 'drink alcohol than those women's partner do not drink alcohol (AOR = 2.19[95%C.I: 1.0, 4..5]) and this is consistent with study conducted in Gondar, Northwest Ethiopia in that physical violence was about five times more likely to occur among women whose male partners consume alcohol frequently and as similarly reported from India, the commonest reasons for violence in household were husband's alcohol addiction [21,31].

The strength of this study is the use of World Health Organization (WHO) instruments for physical violence which is developed enabled to make the comparison of findings with other national and international literatures to be valid. Before conducting this study enumeration of all household was done to identify married women with intimate partners. As limitation outcome was assessed only by the report of respondents. Therefore, under reporting of physical violence was inevitable due to unwillingness of respondents to disclose their personal information. Since this study was a cross sectional it is difficult to establish causes and effect relationships among outcome of interest and explanatory variable. There are also unusual large odds ratio and wide confidence interval observed in this study which might affect the precision. In addition there are also some variables that were not significantly associated with the outcome of interest. This might be due the small sample size to justify the relationships between the explanatory variables and outcome of interest. The observed counts also being small in some of the variables made the odds ratio so large and wide. Therefore any interpretation of this finding within these variables shall take into account the degree of precision. And also all variables associated with physical violence might not be exhaustively investigated. Beside this, since there is limited data in similar set up we are highly obliged to compare some figures with the study with population based survey which might have different circumstance and social, economic and cultural background. Therefore, readers who read this as a reference shall take in to account this as a limitation to the findings.

## Conclusions

The findings of this study indicated substantial prevalence of intimate partner physical violence in the last 12 months and life time. Indicating that one out of every four women was physically abused. One third of currently pregnant women were found to be at a risk of Intimate Partner Violence. Intimate partner violence ranged from moderate to severe violence; slapping, thrown something and Kicking/hit/bit was the most common type of physical violence encountered among women by their male partner, which shows women in refugee camp are at risk of physical violence even during pregnancy time. Being farmer, Muslim, knowing women in the neighborhood whose husband to beat them, being a Muslim, living in abusive environment having drunkard and farmer partner were found to be a significant predictors of experiencing physical violence. Therefore, there is a need to mobilize the community in camp and create awareness about the adverse consequences of partner abuse through gender advocacy and formal and/or informal education, employing information education and communication was crucial. Providing job opportunities as well as educating on use of excessive substances as alcohol, and shortening duration of stay in camp could help reduce the violence. Advocating on difference religions to include education about the effect of conflict in family relation and encourage women equality especially to Kunama and Saho ethnics is important.

Encouraging the victims of physical violence to report to legal bodies and specify the areas in which most violence occurs (abusive environment) and provide security and implementing the existing legal punishment by raising the awareness about the prevalence and consequent intimate partner violence on the women and their children. Further analytic study is needed to explore the relationship between violence and different religions, health-seeking behavior of victims of violence, attitude of men on violence of wife and other types of violence against women.

## Competing interests

The authors declare that they have no competing interests.

## Authors' contributions

GF conceived and designed the study, performed analysis and interpretation of data and critically review the manuscript. MG assisted with the design conception, analysis, and interpretation of data and the draft the manuscript.AG assisted the study design, data analysis and interpretation and critically reviewed the manuscript. All authors read and approved the final manuscript.

## Pre-publication history

The pre-publication history for this paper can be accessed here:

http://www.biomedcentral.com/1471-2458/12/125/prepub
